# Performance of the LHCb RICH detector at the LHC

**DOI:** 10.1140/epjc/s10052-013-2431-9

**Published:** 2013-05-15

**Authors:** The LHCb RICH Collaboration, M. Adinolfi, G. Aglieri Rinella, E. Albrecht, T. Bellunato, S. Benson, T. Blake, C. Blanks, S. Brisbane, N. H. Brook, M. Calvi, B. Cameron, R. Cardinale, L. Carson, A. Contu, M. Coombes, C. D’Ambrosio, S. Easo, U. Egede, S. Eisenhardt, E. Fanchini, C. Fitzpatrick, F. Fontanelli, R. Forty, C. Frei, P. Gandini, R. Gao, J. Garra Tico, A. Giachero, V. Gibson, C. Gotti, S. Gregson, T. Gys, S. C. Haines, T. Hampson, N. Harnew, D. Hill, P. Hunt, M. John, C. R. Jones, D. Johnson, N. Kanaya, S. Katvars, U. Kerzel, Y. M. Kim, S. Koblitz, M. Kucharczyk, D. Lambert, A. Main, M. Maino, S. Malde, N. Mangiafave, C. Matteuzzi, G. Mini’, A. Mollen, J. Morant, R. Mountain, J. V. Morris, F. Muheim, R. Muresan, J. Nardulli, P. Owen, A. Papanestis, M. Patel, G. N. Patrick, D. L. Perego, G. Pessina, A. Petrolini, D. Piedigrossi, R. Plackett, S. Playfer, A. Powell, J. H. Rademacker, S. Ricciardi, G. J. Rogers, P. Sail, M. Sannino, T. Savidge, I. Sepp, S. Sigurdsson, F. J. P. Soler, A. Solomin, F. Soomro, A. Sparkes, P. Spradlin, B. Storaci, C. Thomas, S. Topp-Joergensen, N. Torr, O. Ullaland, K. Vervink, D. Voong, D. Websdale, G. Wilkinson, S. A. Wotton, K. Wyllie, F. Xing, R. Young

**Affiliations:** 1 Sezione INFN di Genova, Genova, Italy; 2 Sezione INFN di Milano Bicocca, Milano, Italy; 3 European Organization for Nuclear Research (CERN), Geneva, Switzerland; 4 H.H. Wills Physics Laboratory, University of Bristol, Bristol, UK; 5 Cavendish Laboratory, University of Cambridge, Cambridge, UK; 6 STFC Rutherford Appleton Laboratory, Didcot, UK; 7 School of Physics and Astronomy, University of Edinburgh, Edinburgh, UK; 8 School of Physics and Astronomy, University of Glasgow, Glasgow, UK; 9 Imperial College London, London, UK; 10 Department of Physics, University of Oxford, Oxford, UK; 11 Syracuse University, Syracuse, NY USA

## Abstract

The LHCb experiment has been taking data at the Large Hadron Collider (LHC) at CERN since the end of 2009. One of its key detector components is the Ring-Imaging Cherenkov (RICH) system. This provides charged particle identification over a wide momentum range, from 2–100 GeV/*c*. The operation and control, software, and online monitoring of the RICH system are described. The particle identification performance is presented, as measured using data from the LHC. Excellent separation of hadronic particle types (*π*, K, p) is achieved.

## Introduction

**Fig. 1:**
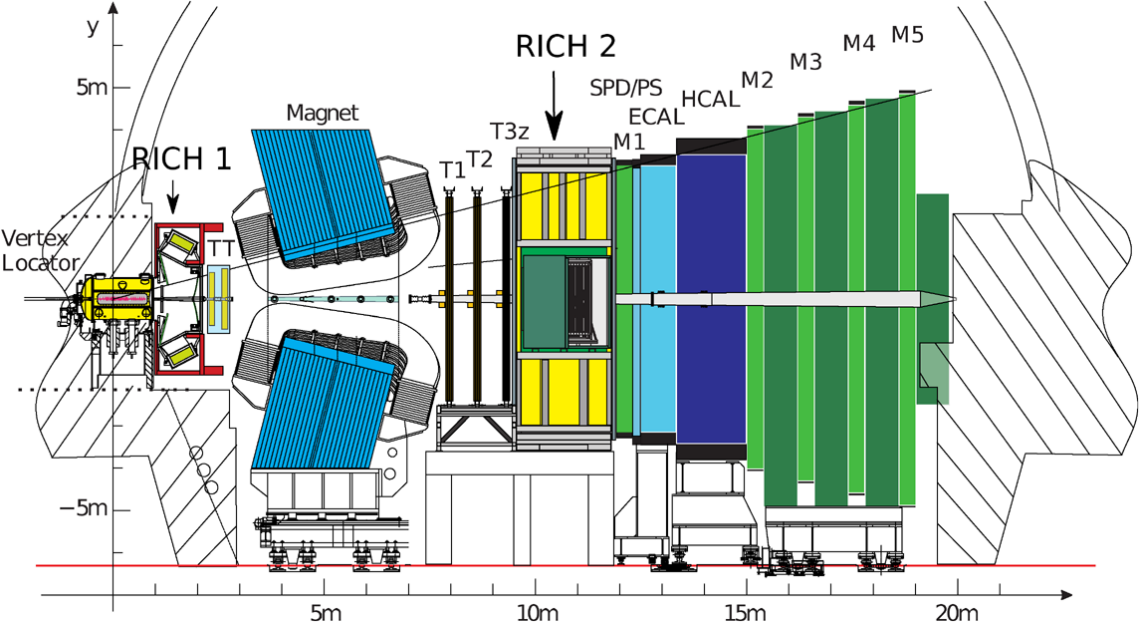
Side view of the LHCb spectrometer, with the two RICH detectors indicated

LHCb [1] is one of the four major experiments at the LHC, and is dedicated to the study of CP violation and the rare decay of heavy flavours. It is a forward spectrometer designed to accept forward-going *b*- and *c*-hadrons produced in proton-proton collisions. The layout of the spectrometer is shown in Fig. 1. The subdetectors of LHCb are described in detail in Ref. [1].

The RICH system of the LHCb experiment provides charged particle identification over a wide momentum range, from 2 to 100 GeV/*c*. It consists of two RICH detectors that cover between them the angular acceptance of the experiment, 15–300 mrad with respect to the beam axis. The LHC accelerator started at the end of 2009 and ran at a centre-of-mass energy of 7 TeV until the end of 2011, followed by 8 TeV in 2012. The luminosity rapidly increased and at the end of 2010 reached the nominal operating value for the LHCb experiment, 2×10^32^ cm^−2^ s^−1^. This paper describes the performance of the RICH system and also its alignment and calibration using data. Many LHCb results have already fully exploited the RICH capabilities [2–5].

The paper is structured as follows: the requirements for particle identification are discussed in Sect. 1, and a brief description of the RICH detectors is given in Sect. 2. The alignment and calibration of the detectors are described in Sect. 3. Section 4 gives an overview of the software used in the RICH reconstruction, particle identification and detector performance, followed in Sect. 5 by the conclusions.

### Requirements for particle identification

The primary role of the RICH system is the identification of charged hadrons (*π*, K, p).

One of the major requirements for charged hadron identification in a flavour-physics experiment is for the reduction of combinatorial background. Many of the interesting decay modes of *b*- and *c*-flavoured hadrons involve hadronic multibody final states. At hadron colliders like the LHC, the most abundant produced charged particle is the pion. The heavy flavour decays of interest typically contain a number of kaons, pions and protons. It is therefore important in reconstructing the invariant mass of the decaying particle to be able to select the charged hadrons of interest in order to reduce the combinatorial background.

**Fig. 2:**
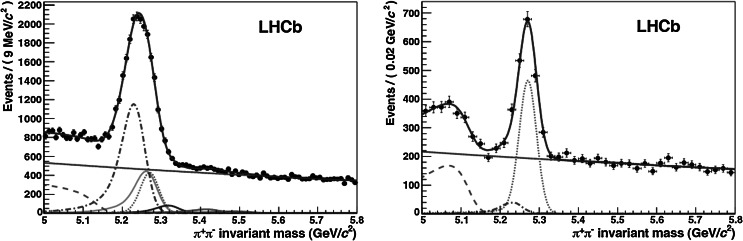
Invariant mass distribution for B→*h* ^+^ *h* ^−^ decays [6] in the LHCb data before the use of the RICH information (*left*), and after applying RICH particle identification (*right*). The signal under study is the decay B^0^→*π* ^+^ *π* ^−^, represented by the turquoise *dotted line*. The contributions from different *b*-hadron decay modes (B^0^→K*π* *red dashed-dotted line*, B^0^→3-body *orange dashed-dashed line*, B_*s*_→KK *yellow line*, B_*s*_→K*π* *brown line*, Λ_*b*_→pK *purple line*, Λ_*b*_→p*π* *green line*), are eliminated by positive identification of pions, kaons and protons and only the signal and two background contributions remain visible in the plot on the right. The *grey solid line* is the combinatorial background (Color figure online)

The second major use of the particle identification information is to distinguish final states of otherwise identical topology. An example is the two-body hadronic decays, B→*h* ^+^ *h* ^−^, where *h* indicates a charged hadron [6]. In this case there are many contributions, as illustrated in Fig. 2, including B^0^→*π* ^+^ *π* ^−^, $\mbox{B}^{0}_{s}\rightarrow \mbox{K}^{+}\mbox{K}^{-}$, and other decay modes of the B^0^, $\mbox{B}^{0}_{s}$ and Λ_*b*_. A signal extracted using only kinematic and vertex-related cuts is a sum over all of the decay modes of this type (Fig. 2 left), each of which will generally have a different CP asymmetry. For a precise study of CP-violating effects, it is crucial to separate the various components. This is achieved by exploiting the high efficiency of the RICH particle identification (Fig. 2 right).

Another application of charged hadron identification is for an efficient flavour tagging [7]. When studying CP asymmetries or particle-antiparticle oscillations, knowledge of the production state of the heavy-flavoured particles is required. This can be achieved by tagging the particle/antiparticle state of the accompanying hadron. Heavy-flavoured particles are predominantly produced in pairs. One of the most powerful means of tagging the production state is by identifying charged kaons produced in the *b*→*c*→*s* cascade decay of the associated particle. Such tagged kaons (as well as kaons from the *b* fragmentation when a $\mbox{B}^{0}_{s}$ is created), have a soft momentum distribution, with a mean of about 10 GeV/*c*. Particle identification down to a few GeV/*c* can therefore significantly increase the tagging power of the experiment.

The typical momentum of the decay products in two-body *b* decays is about 50 GeV/*c*. The requirement of maintaining a high efficiency for the reconstruction of these decays leads to the need for particle identification up to at least 100 GeV/*c*. The lower momentum limit of about 2 GeV/*c* follows from the need to identify decay products from high multiplicity B decays and also from the fact that particles below this momentum will not pass through the dipole magnetic field (4 Tm) of the LHCb spectrometer.

**Fig. 3:**
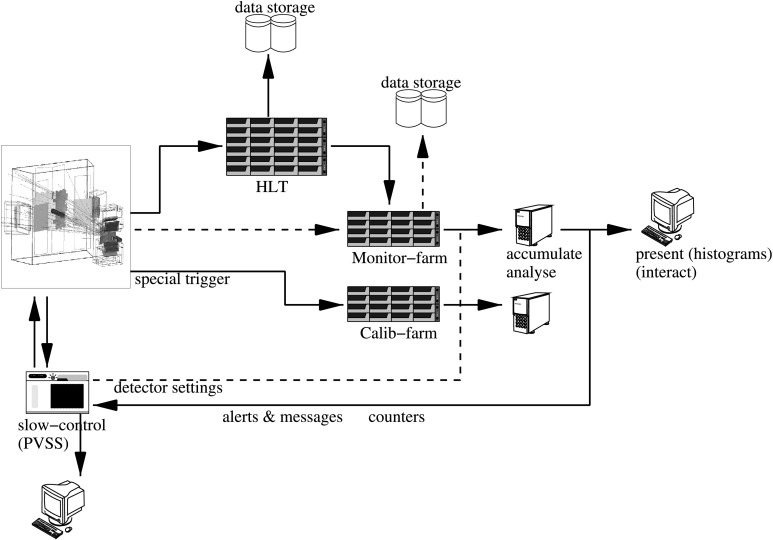
RICH data-flow through the online system. Events selected by the L0 trigger are sent to the High Level Trigger (HLT) farm and, if they pass this trigger requirements, are sent to storage. A fraction of these events (typically 10 %) is also sent to the monitoring farm. Online monitoring algorithms examine the data for irregularities and send messages to the slow-control (ECS) that can trigger automatic actions. Special triggers are sent directly to the calibration farm bypassing the High Level Trigger

A further example of the requirements for particle identification in LHCb is its use in the trigger. LHCb has a high performance trigger system [8], that reduces the event rate from the 40 MHz bunch crossing frequency down to about 2 kHz that can be written to storage. This is achieved in two steps. The first trigger level is implemented in hardware and is based on high transverse energy deposits in the calorimeter and high transverse momentum detected by the muon system, to reduce the rate to 1 MHz. All detectors are then read out into a CPU farm where a high level trigger (HLT, see Fig. 3) decision is made fully in software. The RICH reconstruction is fast enough to contribute to this trigger. An example is the online selection of the *ϕ* particle, which is present in many of the decay modes of interest.

## The RICH detectors

### Detector description

A description of the LHCb RICH detectors is given in Ref. [1]. Only the major features are summarized here. In the forward region, covered by the LHCb spectrometer, there is a strong correlation between momentum and polar angle, with the high-momentum particles produced predominantly at low polar angles. As a result, the RICH system has two detectors. RICH 1 covers the low and intermediate momentum region 2–40 GeV/*c* over the full spectrometer angular acceptance of 25–300 mrad. The acceptance is limited at low angle by the size of the beampipe upstream of the magnet. RICH 2 covers the high-momentum region 15–100 GeV/*c*, over the angular range 15–120 mrad.

To limit its overall volume, RICH 1 is placed as close as possible to the interaction region. It is located immediately downstream of the silicon-microstrip vertex locator (VELO), as shown in Fig. 1. To minimize the material budget there is no separate entrance window, and the RICH 1 gas enclosure is sealed directly to the exit window of the VELO vacuum tank. The downstream exit window is constructed from a low-mass carbon-fibre/foam sandwich. RICH 2 is placed downstream of the magnet, since the high momentum tracks it measures are less affected by the magnetic field. In this way it can be placed after the downstream tracking system in order to reduce material for the measurement of the charged tracks. The entrance and exit windows are again a foam sandwich construction and skinned with carbon-fibre and aluminium, respectively.

Both RICH detectors have a similar optical system, with a tilted spherical focusing primary mirror, and a secondary flat mirror to limit the length of the detectors along the beam direction. Each optical system is divided into two halves on either side of the beam pipe, with RICH 1 being divided vertically and RICH 2 horizontally. The vertical division of RICH 1 was necessitated by the requirements of magnetic shielding for the photon detectors, due to their close proximity to the magnet. The spherical mirrors of RICH 1 (4 segments) are constructed in four quadrants, with carbon-fibre structure, while those of RICH 2 (56 segments), and all flat mirrors (16 and 40 segments in RICH 1 and RICH 2 respectively), are tiled from smaller mirror elements, employing a thin glass substrate. A reflectivity of about 90 % was achieved for the mirrors, averaged over the wavelength region of interest, 200–600 nm. The total material budget for RICH 1 is only about 8 % *X*_0_ within the experimental acceptance, whilst that of RICH 2 is about 15 % *X*_0_.

Fluorocarbon gases at room temperature and pressure are used as Cherenkov radiators; C_4_F_10_ in RICH 1 and CF_4_ in RICH 2 were chosen for their low dispersion. The refractive index is respectively 1.0014 and 1.0005 at 0 ^∘^C, 101.325 kPa and 400 nm. About 5 % CO_2_ has been added to the CF_4_ in order to quench scintillation in this gas [9].

The momentum threshold for kaons to produce Cherenkov light in C_4_F_10_ is 9.3 GeV/*c*. Particles below this momentum would only be identified as kaons rather than pions in veto mode, i.e. by the lack of Cherenkov light associated to the particle. To maintain positive identification at low momentum and in order to separate kaons from protons, a second radiator is included in RICH 1: a 50 mm thick wall made of 16 tiles of silica aerogel [10] at the entrance to RICH 1. The refractive index is *n*=1.03 and the light scattering length is around 50 mm at 400 nm in pure N_2_. The aerogel is placed in the C_4_F_10_ gas volume and a thin glass filter is used on the downstream face to limit the chromatic dispersion.

The Cherenkov photons emitted by charged particles passing through the RICH radiators are focused into ring images on the photon detector planes, situated outside of the spectrometer acceptance. A novel hybrid photon detector (HPD) was developed in collaboration with industry specifically for application in the LHCb RICH system [11]. The HPDs employ vacuum tubes with a 75 mm active diameter, with a quartz window and multialkali photocathode. The photoelectrons are focused onto a silicon pixel array, using an accelerating voltage of −16 kV. The pixel array is arranged in 32 columns and 32 rows, giving a total of 1024 pixels per tube. The pixel size is 2.5×2.5 mm^2^ at the level of the photocathode. A total of 484 HPDs are close-packed to cover the four photodetector planes. Two planes are employed in each RICH, with 196 tubes used in RICH 1 and 288 in RICH 2. The photodetector planes are separated from the radiator gas volumes by quartz windows, and the photodetector volumes are maintained in an atmosphere of CO_2_. The front-end electronics chip is encapsulated within the HPD vacuum tube, and bump-bonded to the silicon pixel sensor, which results in extremely low noise (typically 150 e^−^ RMS per pixel for a signal of 5000 e^−^ [12, 13]). The tubes also feature high detection efficiency, with an active area fraction of about 82 %. The quantum efficiency is about 30 % at 270 nm.

### Detector operation

The operation of the RICH detectors is fully automated and is controlled by the Experiment Control System (ECS) [14–16]. The RICH ECS has been built using components from the Joint Controls Project framework [17], developed by CERN and the four main LHC experiments. The ECS uses predefined sequences for normal detector operation, allowing non-experts to operate the detectors. Automated actions protect the equipment when monitored parameters fall outside the range of accepted values. Sensors are used as input to the LHCb Detector Safety System which put the detectors in a safe state in case of a major malfunction of the control system.

The RICH ECS also collects environmental information that is required by the RICH reconstruction software. There are systems to monitor movements of the RICH mirrors, monitor the quality of the gas radiators, and log the temperature and pressure of the radiators in order to correct the refractive index. Changes in temperature and pressure, which necessitate the re-calculation of the refractive index of the gas radiators, are automatically propagated to the Conditions Database [18].

The RICH detectors and the data recorded are monitored at several stages of the data-acquisition and reconstruction chain to identify any potential problems as early as possible. Figure 3 illustrates the online data-flow, highlighting the dedicated monitoring and calibration farms for analyses using fully reconstructed events.

Low-level processes monitor the data integrity during data recording by cross-checking the various data-banks and reporting any irregularities. Higher-level monitoring algorithms use a neural network [29] to identify Cherenkov rings using information from the RICH detectors only. On rare occasions, an individual HPD may lose synchronisation with the rest of the detector and transmit spurious data for each event. It is found that the performance of the particle identification is affected only marginally by a few units of malfunctioning HPDs,^1^ and it is usually more effective to continue recording data and reset those affected front-end components during the next run initialization. In order to prevent inefficiencies during data-taking due to anomalously busy events, the online monitoring task automatically detects these cases and the read-out electronics discards all data prior to transmission.

Special calibration triggers can be sent to the photodetectors during normal data-taking to activate a pre-defined test pattern of hits. This provides a continuous test of the response of all photodetectors, especially in low-occupancy regions. As these calibration triggers are sent during gaps in the bunch-train structure of the LHC beam, these events contain no activity related to proton-proton interactions. These “empty” events can also be used to evaluate noise that would be present in the detector during data-taking.

The online monitoring allows the full event reconstruction of a sizeable fraction of the events being recorded to be processed online. This allows the monitoring of the reconstructed Cherenkov angle, as well as the alignment of the RICH detectors with respect to the tracking system.

## Alignment and calibration

The tasks of spatial alignment of the RICH detectors and the calibration of the refractive indices of the radiators are performed with data using high momentum charged particles. In addition, the alignment of several mirror segments and the purity of the gas radiators are also monitored using systems that can provide information independently and during periods when there are no collisions.

### Time alignment

In order to maximise the photon collection efficiency of the RICH, the HPD readout must be synchronised with the LHC bunch crossing to within a few nanoseconds. This procedure is referred to as “time alignment” in the following. Individual HPDs vary in timing due to variations in drift time of the electrons within the silicon sensor. HPD readout is triggered by a 25 ns wide strobe pulse distributed by the LHCb network of optic fibres and detected by the RICH Level-0 (L0) boards [19]. A RICH L0 board supervises the triggering, timing and control of the HPDs, with two HPDs serviced by a single board. HPDs that share a L0 board were chosen to have similar timing characteristics in order to optimise the time alignment.

Three features on the timing profile are defined: the *turn*-*on* point is the delay between optimal readout efficiency and the strobe pulse at which 90 % of the peak photon collection efficiency is observed, the *turn*-*off* point is the delay at which the profile drops below 90 %, and the midpoint is the average delay between these values.

**Fig. 4:**
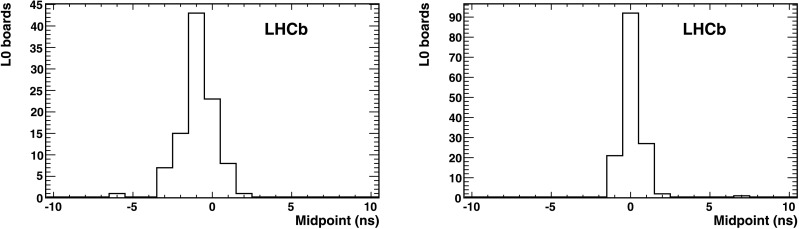
Distribution of the midpoints in RICH 1 (*left*) and RICH 2 (*right*) after time alignment with pp collisions. The RMS deviations of the HPDs are approximately 1 ns

The global time alignment of L0 boards has been performed in several steps both prior to and during running at the LHC. The initial alignment was performed in the absence of beam using a pulsed laser, resulting in a relative alignment of the HPDs in each photodetector plane. The global time alignment to the LHCb experiment is achieved with pp collisions using the LHCb first level trigger. The distribution of the midpoints can be seen in Fig. 4, showing that the HPDs have been time aligned to ∼1 ns.

### Magnetic distortions

Inside an HPD, photoelectrons travel up to 14 cm from the photocathode to the silicon anode. This device is therefore sensitive to stray magnetic fields from the LHCb spectrometer magnet. All HPDs in RICH 1 and RICH 2 experience some residual fringe field: the magnetic shields surrounding the HPDs reduce it from initial values of up to 60(15) mT in RICH 1(RICH 2), to a maximum value of ≃2.4 mT in RICH 1, and values ranging between 0.2–0.6 mT in RICH 2. The resulting distortion of the images are mapped and corrected when reconstructing Cherenkov angles.

A characterisation procedure has been developed to correct for magnetic distortion effects and restore the optimal resolution. Different implementations are used for RICH 1 and RICH 2 as the two detectors have different geometries and experience different field configurations.

#### RICH 1

The distortions of the HPD images are corrected using a dedicated calibration system. The mapping system consists of two identical hardware arrangements, one for each of the upper and lower HPD boxes [20]. Each system consists of an array of green light-emitting diodes mounted on a carbon-fibre support that spans the width of the HPD box. This “light bar” attaches at each end to a pair of synchronised stepper-motors that facilitates the illumination of the entire HPD array. The light bar comprises 19 LED units each with an array of 5×28 LEDs, 2.5 mm apart. A passive collimator unit is mounted flush to the LED array such that light from each LED is channeled down an individual collimator with 0.3 mm aperture.

The distortion is mapped by comparing the pattern of light spots in magnet-on and magnet-off data. The direction of the magnetic field is predominantly longitudinal with respect to the tube axis. The field effect causes a rotation of the image about the central axis of the HPD, and a modest expansion of the image. The residual transverse component of the field displaces the centre of the photocathode image.

**Fig. 5:**
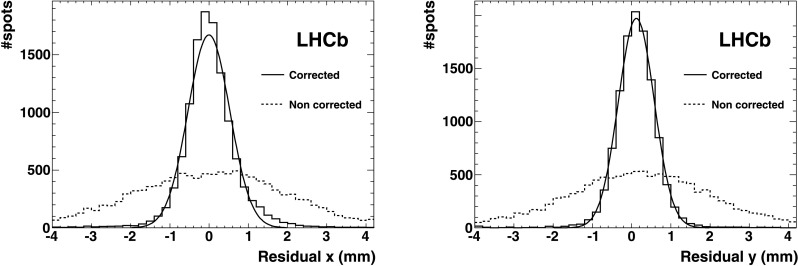
Spatial residuals demonstrating the resolution with which the light spots of the test pattern in RICH 1 are identified. The plot shows the distance from the measured light spot centre to the nearest test point. The *dotted* and *solid lines* are before and after the calibration respectively, along the *x* direction (*left*) and along *y* (*right*) of the anode plane, projected on the photocathode plane. The *solid line* is the Gaussian fit (Color figure online)

The result of the parametrisation is demonstrated in Fig. 5, showing the residual positional uncertainty due to the magnetic distortion after the correction procedure. The resolution after correction is *σ*≃0.2 pixel (0.5 mm), significantly smaller than the irreducible uncertainty of *σ*≃0.29 pixel (0.72 mm), originating from the finite HPD pixel size.

#### RICH 2

The magnetic distortion correction for RICH 2 uses a system based on the projection of a light pattern onto the plane of HPDs using a commercial light projector. The projected pattern is a suitable grid of light spots. The algorithm to reconstruct the position of a light spot builds a cluster of hits and the cluster centre is calculated. A resolution better than the pixel size is achieved. Comparing the position of the light spots with and without the magnetic field makes it possible to measure and parametrise the magnetic field distortions [21, 22].

**Fig. 6:**
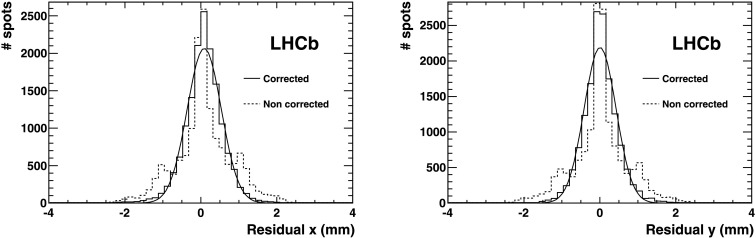
Spatial residuals demonstrating the resolution with which the light spots of the test pattern in RICH 2 are identified. The plot shows the distance from the measured light spot centre to the nearest test point. The *dotted* and *solid lines* are before and after the calibration respectively. Most of the photodetectors of RICH 2 are in a region free from magnetic field residual values (region around *x*=0 of the *dotted histogram*). Where these are different from zero, the distortions induced are visible in the two satellite peaks of opposite sign (the magnetic field changes sign in the *upper* and *lower* part of the photodetector matrix plane). The *left plot* is the measurement along the *x*, the *right plot* along *y* of the anode plane, projected on the photocathode plane. The *solid line* is the Gaussian fit

The distortion mainly consists of a small rotation (on average ≲0.1 rad) of the test spots around the HPD axis. This rotation varies from HPD to HPD, depending on the HPD position. No measurable variation of the radial coordinate of the light spots was detected. The parameters extracted using either orientation of the magnetic field also apply, with a sign inversion, to the opposite magnetic field polarity. By applying the correction procedure, the resolution on the position of the light spot improves from *σ*≃0.33 pixel to *σ*≃0.19 pixel (0.47 mm) (see Fig. 6). For comparison the pixel size resolution is *σ*=0.29 pixel (0.72 mm).

### Detector alignment

In order to reconstruct the Cherenkov angle associated with the individual photons as accurately as possible, a number of components must be aligned with an accuracy of 0.1 mrad with respect to the LHCb tracking system. The alignment procedure calculates the misalignments of the various detector components in a sequential process. First, the whole RICH detector is aligned with the global LHCb coordinate system, followed by each detector half, each mirror segment and finally each HPD. This includes aligning the silicon sensors inside the HPDs to the whole RICH detector. One has to know the position of the centre of each HPD photocathode on the anode. The silicon sensors are aligned by mapping an image of the photocathode. This procedure does not require the reconstruction of the Cherenkov angle. The relative HPD alignment can also be corrected using data from the magnetic distortion measurements. After these steps, the alignment procedure uses the reconstructed Cherenkov angle of *β*≈1 particles to align the whole RICH detector, the HPD panels, and eventually the 4 (56) spherical and 16 (40) flat mirror segments in RICH 1 (RICH 2).

**Fig. 7:**
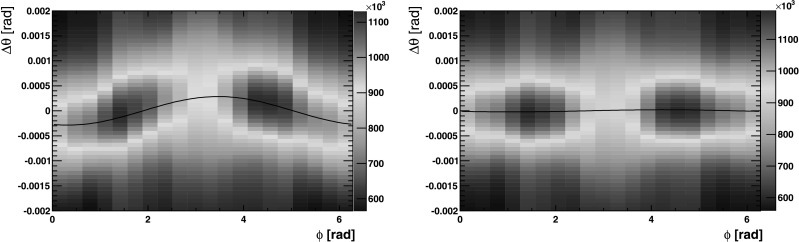
Δ*θ*_*C*_ plotted as a function of the azimuthal angle *ϕ* and fitted with *θ*_*x*_cos(*ϕ*)+*θ*_*y*_sin(*ϕ*), for one side of the RICH 2 detector. The *left-hand plot* is prior to alignment, and shows a dependency of the angle *θ*_*C*_ on the angle *ϕ*. The *right-hand plot* is after the alignment correction, and Δ*θ*_*C*_ is uniform in *ϕ*

Any misalignment of the RICH detectors with respect to the tracking system is observed as a shift of the track projection point on the photodetector plane from the centre of the corresponding Cherenkov ring. This shift is observed by analysing the Cherenkov angle, *θ*_*C*_, as a function of the azimuthal Cherenkov angle *ϕ*, defined as the angle of the pixel hit in the coordinate system of the photodetector plane, with the projected track coordinate at the origin. The angle *θ*_*C*_ is independent of the angle *ϕ* for a well aligned detector, whilst a misaligned system would result in a sinusoidal distribution as shown in Fig. 7.

In practice, distributions of Δ*θ*_*C*_ against *ϕ* are plotted where Δ*θ*_*C*_=*θ*_*C*_−*θ*_0_ and *θ*_0_ is the Cherenkov angle calculated from the momentum of the track and the refractive index of the radiator. Any systematic shift away from the value *θ*_0_ is observable as a shift in Δ*θ*_*C*_.

The Δ*θ*_*C*_ distribution is then divided into slices in *ϕ*. For each slice, a one dimensional histogram of Δ*θ*_*C*_ is fitted with a Gaussian plus a second order polynomial background and the peak of the distribution is extracted. The mean of each slice fit is then used to fit a sinusoidal distribution given by

$$\Delta \theta_C = \theta_{x} \cos(\phi) + \theta_{y}\sin(\phi). $$

The final fit is shown in Fig. 7; the extracted values of *θ*_*x*_ and *θ*_*y*_ correspond to a misalignment on the HPD detector plane in the *x* and *y* direction respectively.

The alignment of the mirror segments has the extra complication that every photon is reflected twice, and so the data must be separated into samples which have unique spherical and flat mirror combinations. For this procedure, only photons that can be uniquely associated to a given mirror pair are used. Mirror segments are identified by considering photons to have been emitted at both the start and end of the gas radiators. If the mirror segments reflecting the photons are the same in both cases, the photon trajectory is considered unambiguous and is used for the alignment of mirror segments.

The mirror arrangement in RICH 1 allows for alignment using a sequential approach as described above, where the spherical mirrors are aligned first, followed by the planar mirrors. This is possible because photons reaching a particular planar mirror can only be reflected from a single spherical mirror [23]. In RICH 2 the larger number of spherical/planar mirror combinations makes the use of a sequential method impossible. The alignment of the RICH 2 mirror segments is performed by solving a set of simultaneous equations to extract all the alignment parameters of all the mirrors. One iteration of this method is required to obtain the final mirror alignment.

### Refractive index calibration

The refractive index of the gas radiators depends on the ambient temperature and pressure and the exact composition of the gas mixture. It can therefore change in time, and this affects the performance of the particle identification algorithms. The ultimate calibration of the refractive index is performed using high momentum charged particle tracks in such a way that the distribution of Δ*θ*_*C*_ peaks at zero.

The calibration of the refractive index of the aerogel is performed using tracks with momentum *p*>10 GeV/*c* passing through each tile. It is found not to change as a function of time.

### Monitoring hardware

There are additional monitoring tasks, independent from the methods described above.

The four spherical mirrors in RICH 1 and 20 of the mirror segments in RICH 2 are monitored for stability using laser beams and cameras. For each monitored mirror there is an optic fibre with a lens to provide a focused beam, a beam splitter, a mirror and a camera. The beam splitter creates two beams. The reference beam is incident directly onto the camera. The second beam is reflected to the camera via the monitored mirror. A comparison of the relative position of these light spots tracks possible movement of the mirror.

The purity of the gas radiators is monitored by measuring the speed of sound in the gas. A 50 kHz ultrasonic range finder is used. The gas to and from the detector is monitored with a precision of about 1 % for a binary gas mixture. A gas chromatograph is periodically used for high precision measurements. Any variation in time, after correction for temperature effects, is likely due to changes in the composition of the gas.

**Fig. 8:**
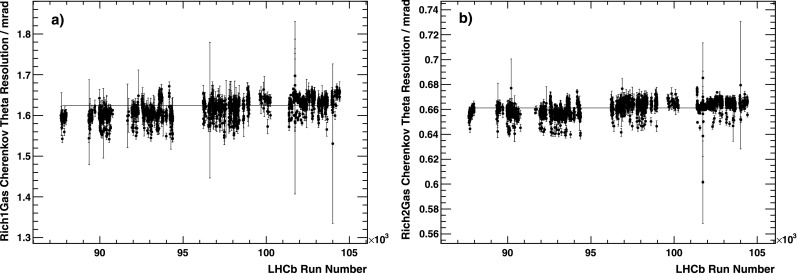
The Cherenkov angular resolution (cf. Sect. 4.2), after all corrections have been applied, as a function of run number. (**a**) For RICH 1 and (**b**) for RICH 2. The period of time covered on the *x*-axis corresponds to about 8 months of running

After correcting for all the parameters monitored as a function of time as described in this section, the detector behaviour is very stable, as shown in Fig. 8.

## Performance

### Data reconstruction

The LHCb software is based on the Gaudi Framework [24, 25] which provides a flexible and configurable C++ Object Oriented framework. This flexibility allows the same software to be used in a variety of different RICH applications, ranging from the online monitoring, the utilization of the RICH in the final stages of the higher level trigger, and providing the full offline event reconstruction. This section describes the processing steps of the RICH data.

#### HPD data reconstruction

The first stage of the data processing chain is to decode the raw data, as read out from the detector, to offline storage. This produces a list of the HPD pixels that have been hit in each event. The next step is to apply various data cleaning algorithms to the list of active pixels for each HPD. HPD data are rejected if the HPD occupancy, which on average is ∼1 %, exceeds a tuneable maximum value of 20 %, to exclude excessively large events.

Finally, the position of the photon hit is reconstructed on the HPD plane. This procedure corrects for the alignment of the HPDs within the LHCb detector, the electrostatic focusing parameters of the HPD tubes, and the corrections for the magnetic field (Sect. 3.2).

#### Cherenkov photon candidate reconstruction

The tracking system of LHCb provides detailed coordinate information on the passage of charged particles through the LHCb spectrometer, and with this information the trajectory of each particle through the three RICH radiator volumes can be determined. This allows the computation of an assumed emission point of the photon candidates for each track. As the exact emission point of each photon is unknown (and can be anywhere along the particle trajectory through the radiator), the mid-point of the trajectory in the radiator is taken.

The candidate photons for each track are determined by combining the photon emission point with the measured hit positions of the photons. Once the photon candidates have been assigned, quantities such as the Cherenkov angle *θ*_*C*_, can be computed. A full analytical solution of the RICH optics is used, which reconstructs the trajectory of the photon through the RICH optical system, taking into account the knowledge of the mirror and HPD alignment [26].

### Cherenkov angle resolution

**Fig. 9:**
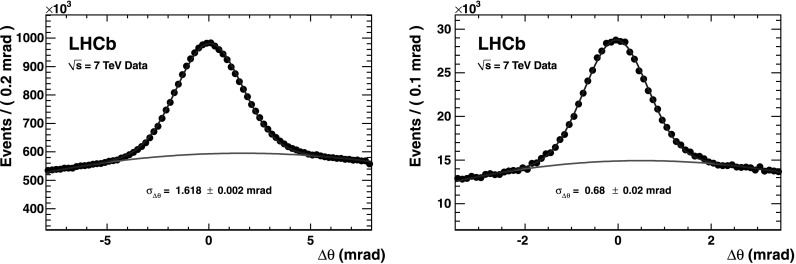
Single photoelectron resolution for the RICH 1 (*left*) and RICH 2 (*right*) gases, as measured in data for high momentum charged particles. The *red line* describes the background as determined from the fit using a polynomial function together with the Gaussian for the signal (Color figure online)

The distribution of Δ*θ*_*C*_, calculated for each photon with respect to the measured track, is shown for the RICH 1 and RICH 2 gas radiators in Fig. 9 after the alignment and calibration procedures have been performed.

By fitting the distribution with a Gaussian plus a polynomial background, the Cherenkov angle resolution is determined to be 1.618±0.002 mrad for C_4_F_10_ and 0.68±0.02 mrad for CF_4_. These values are in reasonable agreement with the expectations from simulation [27, 28] of 1.52±0.02 mrad and 0.68±0.01 mrad in RICH 1 and RICH 2, respectively.

**Fig. 10:**
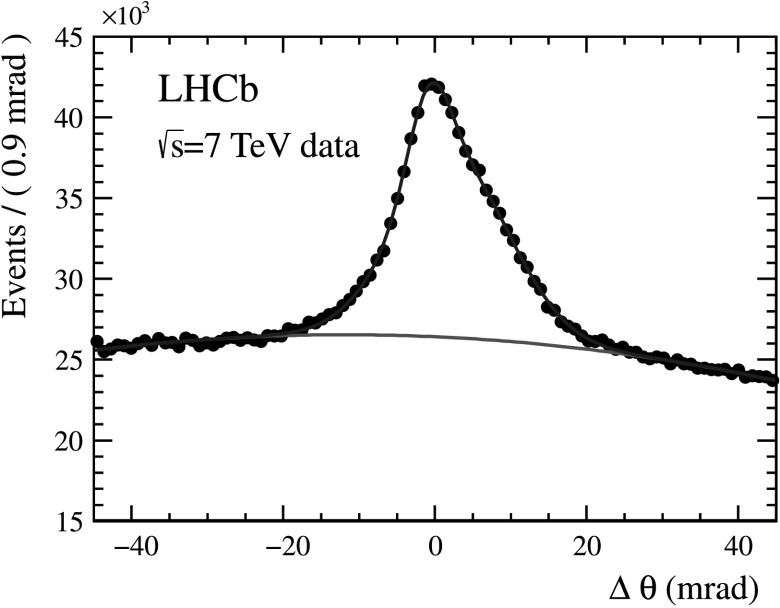
Single photoelectron resolution for the aerogel as measured in 2011 data with the pp→pp*μ* ^+^ *μ* ^−^ events. The *red line* describes the background as determined from the fit using a polynomial function together with two Gaussians for the signal

The performance of the aerogel radiator has been studied with data collected in 2010 and 2011. The data have been first used to calibrate the refractive indices of individual tiles. Figure 10 shows the deviation Δ*θ*_*C*_ in the four aerogel tiles located around the beampipe, which cover more than 90 % of the acceptance. The Δ*θ*_*C*_ distribution of the photons is measured using good quality tracks with momentum above 10 GeV/*c*. The peak is not symmetric, and the *σ* from the FWHM gives an average value of about 5.6 mrad (the events used for this estimate are all pp collisions, not the ones used in Fig. 10). This value is about a factor of 1.8 worse than the simulation. This discrepancy is, at least partially, explained by the absorption by the very porous aerogel structure of the C_4_F_10_ with which it is in contact.

A new aerogel enclosure which isolates the aerogel from the C_4_F_10_ gas in RICH 1 is installed for the 2012 running.

### Photoelectron yield

The photoelectron yield N$_{\rm pe}$ is measured for two categories of RICH event: one, referred to as a *normal* event, is representative of nominal RICH running conditions during LHCb physics data collection; the other, referred to as an *ideal* event, is a special event type with very low photoelectron backgrounds and clean tracks with full, unobstructed Cherenkov rings.

The *normal* event category uses an unbiased (in that the RICH detectors are not used in the selection) track sample composed of kaons and pions originating from the decay D^0^→K^−^ *π* ^+^, where the D^0^ is selected from D^∗+^→D^0^ *π* ^+^ decays. The kaons and pions are required to have track momenta $p_{\rm K}>9.8~\mathrm{GeV}/c$ and *p*_*π*_>5 GeV/*c* in the aerogel; $p_{\rm K}>37~\mathrm{GeV}/c$ and *p*_*π*_>30 GeV/*c* in C_4_F_10_, and $p_{\rm K}>74.8~\mathrm{GeV}/c$, *p*_*π*_>40.4 GeV/*c* in CF_4_. These cuts ensure that all tracks have an expected Cherenkov angle close to saturation (*β*≈1).

The track sample of the *ideal* event category is composed of muons selected from pp→pp*μ* ^+^ *μ* ^−^ events. The events are required not to have a visible primary vertex. The track momentum selection criteria of the muons is the same as for pions in the *normal* event category. A cut was applied on the track geometry, such that at least half of the Cherenkov cone associated to the track projects onto the HPD pixels. This selection avoids losses owing to the cone intersecting with the beampipe, or projecting onto the region outside the HPD acceptance and the gaps between the HPDs.

**Fig. 11:**
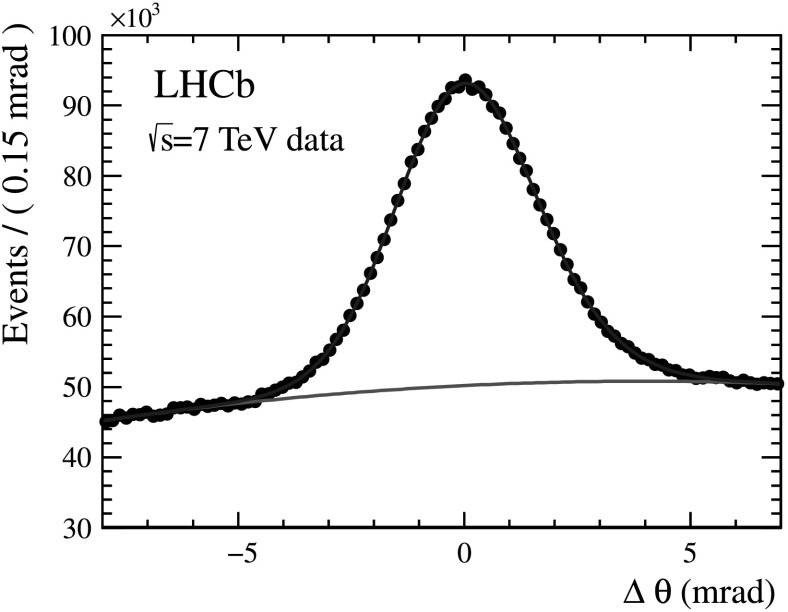
Distribution of Δ*θ*_*C*_ for C_4_F_10_. This plot is produced from kaons and pions from tagged D^0^→K^−^ *π* ^+^ decays in data selected with the criteria described in the text

N$_{\rm pe}$ is measured by fitting the Δ*θ*_*C*_ distributions of the photoelectrons. For each selected charged particle track, photoelectron hits that lie within a Δ*θ*_*C*_ range of ±5*σ*, where *σ* is the Cherenkov angle resolution, are retained. Photoelectrons that are correctly associated with a track peak around Δ*θ*_*C*_=0 and are distributed as a Gaussian, while those from other tracks and background sources form a non-peaking background, as shown in Fig. 11 obtained from C_4_F_10_.

**Fig. 12:**
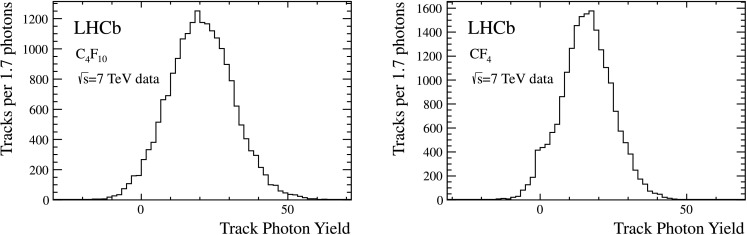
Individual track photon yield distributions for the C_4_F_10_ (*left*) and CF_4_ (*right*) radiators. The plot is produced from kaons and pions from tagged D^0^→K^−^ *π* ^+^ decays in data selected with the criteria described in the text

An initial fit is performed on the Δ*θ*_*C*_ distribution aggregated from all the selected tracks, using a probability density function (PDF) composed of a Gaussian signal over a quadratic background. The Δ*θ*_*C*_ distribution of each individual track is then fitted with a Gaussian signal over a linear background PDF, with the mean of the Gaussian fixed at 0 and the width fixed to that obtained from the fit to the aggregated Δ*θ*_*C*_ distribution. The individual track N$_{\rm pe}$ is then taken as the number of photoelectron candidates under the Gaussian shape. The overall value for N$_{\rm pe}$ is taken as the mean of the distribution of the track N$_{\rm pe}$, with the error corresponding to the standard error on the mean. Figure 12 shows the data distributions at the basis of the measurement.

The validity of the N$_{\rm pe}$ calculation method was assessed using simulated samples of D^∗+^→D^0^(K^−^ *π* ^+^)*π* ^+^ decays. The same selection criteria were applied as in data and in addition the track geometry selection was applied with the same criteria as for *ideal* RICH events, to allow a like-for-like comparison between simulation and *ideal* data events. The calculated value for N$_{\rm pe}$ was compared to the true photoelectron yield, which was taken by counting the number of photons associated to each track by the simulation and then taking the average over all tracks.

To allow a like-for-like comparison of the true and calculated N$_{\rm pe}$ values in the simulation study, events were required to have less than 50 hits in the Scintillator Pad Detector (SPD) [1], which gives an approximate measurement of the charged track multiplicity in the event. It has been observed that the measured N$_{\rm pe}$ is lower for high track multiplicity events, which have high HPD occupancies (more than 20 % in the central HPD’s in RICH 1 for events with >500 charged tracks). This results in a loss of detected photoelectrons, because instances where multiple photons hit the same pixel result in only one photoelectron hit due to the binary HPD readout. This suppression of N$_{\rm pe}$ was not observed when an analog HPD readout was emulated in the simulation.

Table 1 shows the results of the analysis performed on real data and on the simulation. In the simulated data, the calculated and true values of N$_{\rm pe}$ are in good agreement for all the radiators. This validates the accuracy of the yield calculation. The N$_{\rm pe}$ values for the *ideal* events are less than those from the simulation sample. The *normal* events have values of N$_{\rm pe}$ that are less than those for *ideal* events. This is mainly due to the higher charged track multiplicities of the *normal* events, reducing the N$_{\rm pe}$, and the track geometry cut that is applied to the *ideal* events increasing their N$_{\rm pe}$ yield. The aerogel N$_{\rm pe}$ data values have a large uncertainty due to the large background in the Δ*θ*_*C*_ distributions and the additional uncertainty in the shape of the signal peak.

**Table 1:** Comparison of photoelectron yields (N$_{\rm pe}$) determined from D^∗^→D^0^ *π* ^+^ decays in simulation and data, and pp→pp*μ* ^+^ *μ* ^−^ events in data, using the selections and methods described in the text

Radiator	N_pe_ from data		N_pe_ from simulation	
	Tagged D^0^→K^−^ *π* ^+^	pp→pp*μ* ^+^ *μ* ^−^	Calculated N_pe_	True N_pe_
Aerogel	5.0±3.0	4.3±0.9	8.0±0.6	6.8±0.3
C_4_F_10_	20.4±0.1	24.5±0.3	28.3±0.6	29.5±0.5
CF_4_	15.8±0.1	17.6±0.2	22.7±0.6	23.3±0.5

The photoelectron yields are lower than those predicted by the simulation: however, there is evidence that the yield in data can be increased by a few percent in RICH 1 by retuning the setting of the HPD readout chip. This retuning was found necessary for all HPDs by the fact that the trigger rate went up significantly during 2011 running, resulting in a readout inefficiency. Furthermore, the detailed description of the detector in the simulation needs continuous retuning, especially for a RICH detector where the Cherenkov photons must interact with many detector elements. It must be stressed however, that the smaller yield measured in data does not have a consequence on the final particle identification performance, as described in Sect. 5.4.

## Particle identification performance

Determining the performance of the RICH Particle IDentification (PID), both during and after data taking, is particularly important for analyses that exploit RICH PID, for which knowledge of efficiency and misidentification rates are required. Moreover, it enables comparison with expectations and provides a benchmark against which to compare the effectiveness of alignment and calibration procedures.

This section provides a description of the PID algorithms and the performance obtained following analysis of data from the first LHC runs.

### Particle identification algorithms

**Fig. 13:**
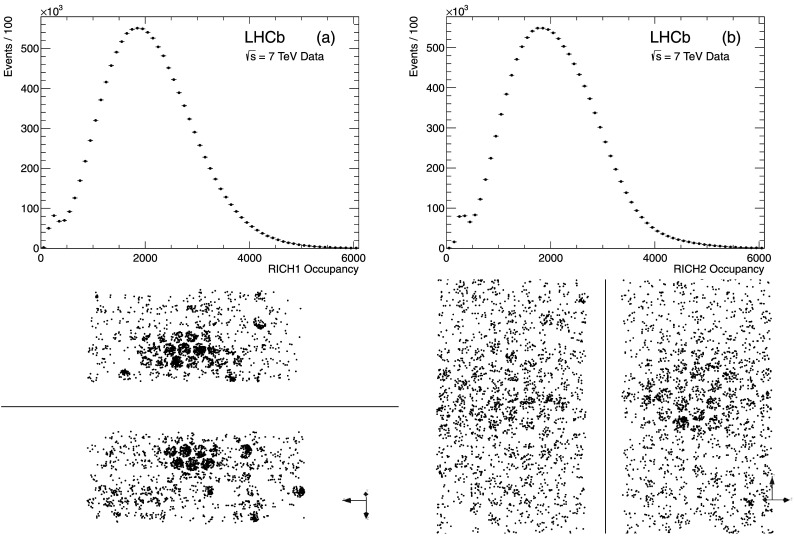
Distribution of the number of pixel hits per event in (**a**) RICH 1 and (**b**) RICH 2. An example of a typical LHCb event as seen by the RICH detectors, is shown below the distributions. The *upper*/*lower* HPD panels in RICH 1 and the *left*/*right* panels in RICH 2 are shown separately

In order to determine the particle species for each track, the Cherenkov angle information must be combined with the track momentum measured by the tracking system, as described in Sect. 4.1.2. The RICH detectors operate in a high occupancy environment, as shown in Fig. 13. To reconstruct such events efficiently, an overall event log-likelihood algorithm is employed, where all tracks in the event and in both RICH detectors are considered simultaneously [26]. This allows for an optimal treatment of tracks where Cherenkov cones overlap.

Since the most abundant particles in pp collisions are pions, the likelihood minimisation procedure starts by assuming all particles are pions. The overall event likelihood, computed from the distribution of photon hits, the associated tracks and their errors, is then calculated for this set of hypotheses. Then, for each track in turn, the likelihood is recomputed changing the mass hypothesis to e, *μ*, *π*, K and proton, whilst leaving all other hypotheses unchanged. The change in mass hypothesis amongst all tracks that gives the largest increase in the event likelihood is identified, and the mass hypothesis for that track is set to its preferred value. This procedure is then repeated until all tracks have been set to their optimal hypotheses, and no further improvement in the event likelihood is found.

The procedure described above is CPU intensive for a large number of tracks and HPD pixels, since the number of likelihood calculations increases exponentially with the number of tracks. In order to counter this, some modifications are made to the minimisation procedure to limit the number of combinations, whilst still converging on the same global solution. During the search for the track with the largest improvement to the event likelihood, the tracks are sorted according to the size of their likelihood change from the previous step, and the search starts with the track most likely to change its hypothesis. If the improvement in the likelihood for the first track is above a tuneable threshold, the search is stopped and the hypothesis for that track is changed. Secondly, if a track shows a clear preference for one mass hypothesis, then once that track has been set to that hypothesis, it is removed in the next iterations. These modifications to the likelihood minimisation dramatically reduce the CPU resources required.

The background contribution to the event likelihood is determined prior to the likelihood algorithm described above. This is done by comparing the expected signal in each HPD, due to the reconstructed tracks and their assigned mass hypothesis, to the observed signal. Any excess is used to determine the background contribution for each HPD and is included in the likelihood calculation.

The background estimation and likelihood minimisation algorithms can be run multiple times for each event. In practice it is found that only two iterations of the algorithms are needed to get convergence. The final results of the particle identification are differences in the log-likelihood values ${ \Delta \log \mathcal{L}}$, which give for each track the change in the overall event log-likelihood when that track is changed from the pion hypothesis to each of the electron, muon, kaon and proton hypotheses. These values are then used to identify particle types.

### Performance with isolated tracks

A reconstructed Cherenkov ring will generally overlap with several others. Solitary rings from *isolated* tracks provide a useful test of the RICH performance, since the reconstructed Cherenkov angle can be uniquely predicted. A track is defined as *isolated* when its Cherenkov ring does not overlap with any other ring from the same radiator.

**Fig. 14:**
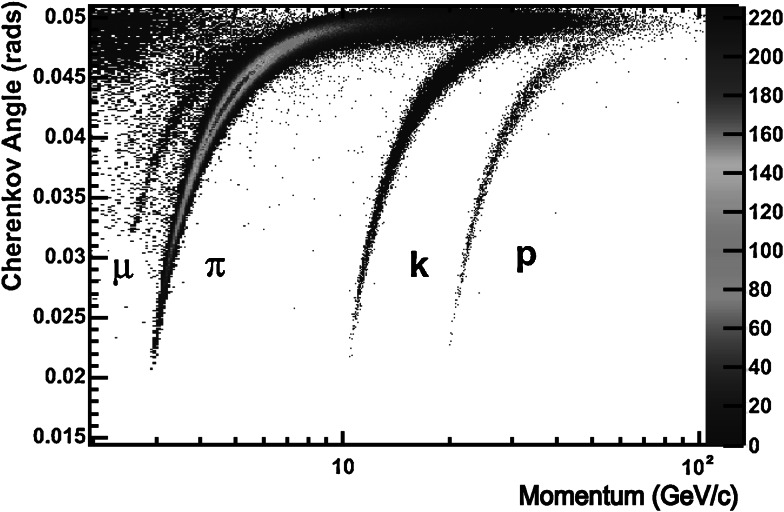
Reconstructed Cherenkov angle as a function of track momentum in the $\rm C_{4}F_{10}$ radiator

Figure 14 shows the Cherenkov angle as a function of particle momentum using information from the C_4_F_10_ radiator for isolated tracks selected in data (∼2 % of all tracks). As expected, the events are distributed into distinct bands according to their mass. Whilst the RICH detectors are primarily used for hadron identification, it is worth noting that a distinct muon band can also be observed.

### PID calibration samples

In order to determine the PID performance on data, high statistics samples of genuine K^±^,*π* ^±^, p and $\bar{\rm p}$ tracks are needed. The selection of such control samples must be independent of PID information, which would otherwise bias the result. The strategy employed is to reconstruct, through purely kinematic selections independent of RICH information, exclusive decays of particles copiously produced and reconstructed at LHCb.

**Fig. 15:**
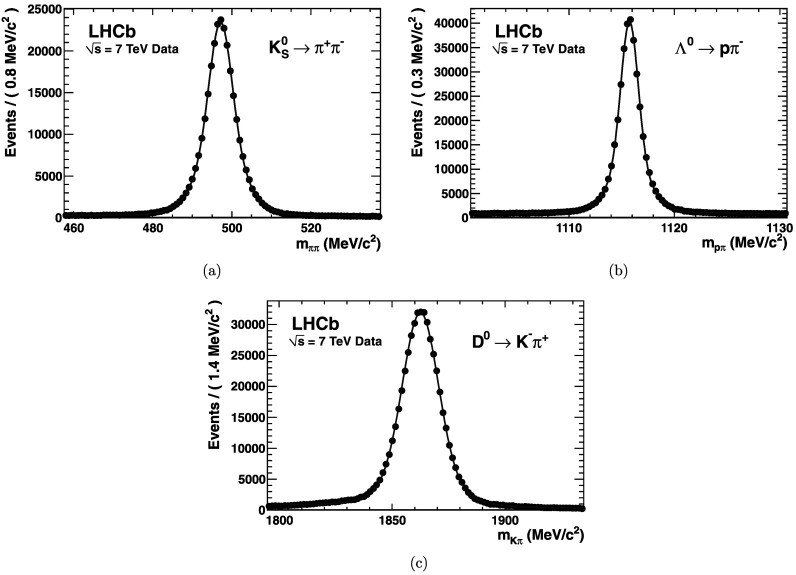
Invariant mass distributions of the (**a**) $\mbox{K}^{0}_{S}$, (**b**) Λ and (**c**) D^0^ calibration samples. The best fit probability-density-function (pdf), describing both background and signal, is superimposed in *blue*

The following decays, and their charge conjugates, are identified: $\mbox{K}^{0}_{\rm S}\,{\to}\,\pi^{+} \pi^{-}$, Λ → p*π* ^−^, D^∗+^→D^0^(K^−^ *π* ^+^)*π* ^+^. This ensemble of final states provides a complete set of charged particle types needed to comprehensively assess the RICH detectors hadron PID performance. As demonstrated in Fig. 15, the $\mbox{K}^{0}_{\rm S}$, Λ, and D^∗^ selections have extremely high purity.

While high purity samples of the control modes can be gathered through purely kinematic requirements alone, the residual backgrounds present within each must still be accounted for. To distinguish background from signal, a likelihood technique, called $_{s}\mathcal{P}\mathit{lot}$ [30], is used, where the invariant mass of the composite particle $\mbox{K}^{0}_{\rm S}, \Lambda$, D^0^ is used as the discriminating variable.

**Fig. 16:**
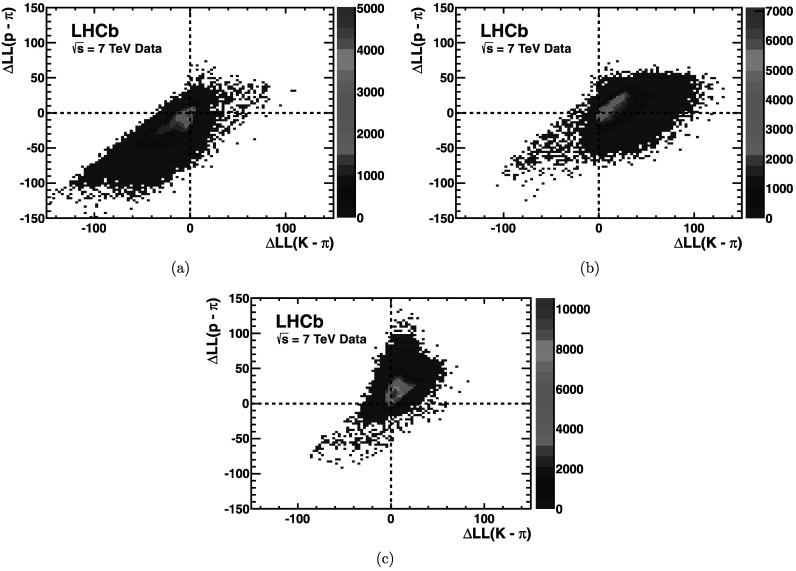
Distribution of $\Delta \log \mathcal{L}(\mbox{K} - \pi)$ against $\Delta \log \mathcal{L}(\mbox{p} - \pi)$ for (**a**) pions, (**b**) kaons and (**c**) protons extracted from the control samples

The power of the RICH PID can be appreciated by considering the $\Delta \log \mathcal{L}$ distributions for each track type from the control samples. Figures 16(a–c) show the corresponding distributions in the 2D plane of $\Delta \log \mathcal{L}(\mbox{K} -\pi)$ versus $\Delta \log \mathcal{L}(\mbox{p} - \pi)$. Each particle type is seen within a quadrant of the two dimensional $\Delta \log \mathcal{L}$ space, and demonstrates the powerful discrimination of the RICH.

### PID performance

**Fig. 17:**
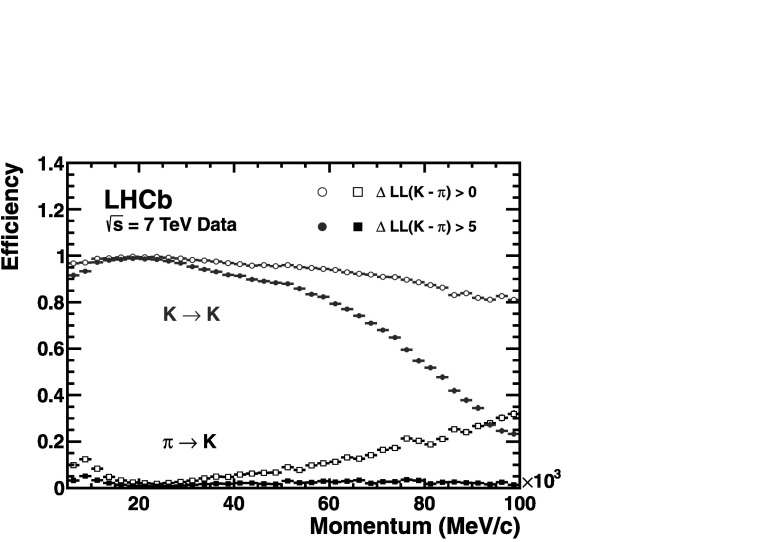
Kaon identification efficiency and pion misidentification rate measured on data as a function of track momentum. Two different $\Delta \log \mathcal{L}(\mbox{K}-\pi)$ requirements have been imposed on the samples, resulting in the open and filled marker distributions, respectively

**Fig. 18:**
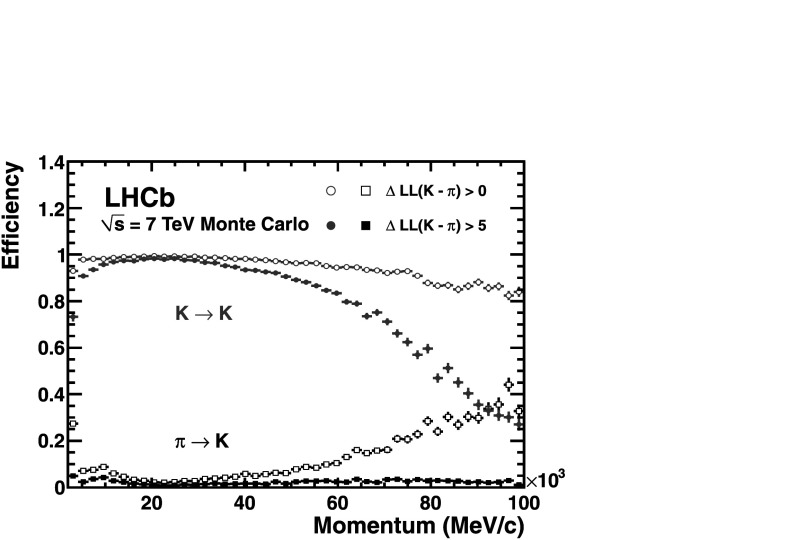
Kaon identification efficiency and pion misidentification rate measured using simulated events as a function of track momentum. Two different $\Delta \log \mathcal{L}(\mbox{K}-\pi)$ requirements have been imposed on the samples, resulting in the open and filled marker distributions, respectively

**Fig. 19:**
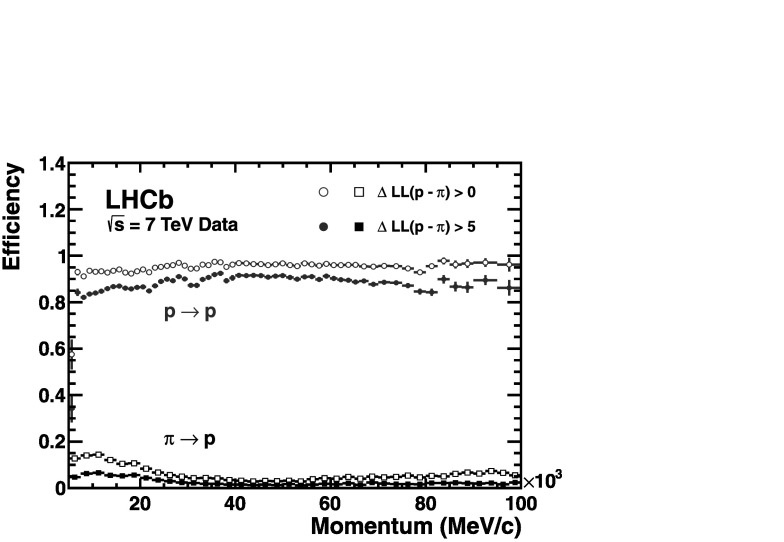
Proton identification efficiency and pion misidentification rate measured on data as a function of track momentum. Two different $\Delta \log \mathcal{L}(\mbox{p}-\pi)$ requirements have been imposed on the samples, resulting in the open and filled marker distributions, respectively

**Fig. 20:**
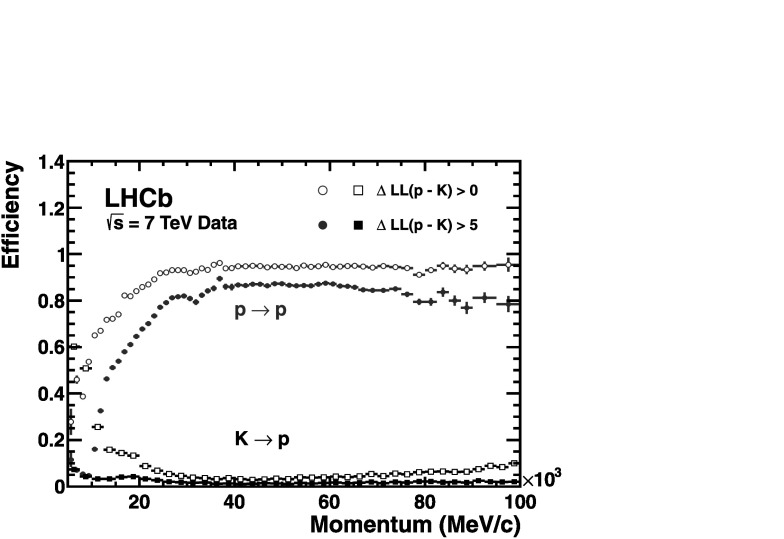
Proton identification efficiency and kaon misidentification rate measured on data as a function of track momentum. Two different $\Delta \log \mathcal{L}(\mbox{p}-\mbox{K})$ requirements have been imposed on the samples, resulting in the open and filled marker distributions, respectively

Utilizing the log-likelihood values obtained from the control channels, one is able to study the discrimination achievable between any pair of track types by imposing requirements on their differences, such as Δlog(K−*π*). Figure 17 demonstrates the kaon efficiency (kaons identified as kaons) and pion misidentification (pions misidentified as kaons), as a function of particle momentum, obtained from imposing two different requirements on this distribution. Requiring that the likelihood for each track with the kaon mass hypothesis be larger than that with the pion hypothesis, i.e. $\Delta \log \mathcal{L}(\mbox{K}-\pi)>0$, and averaging over the momentum range 2–100 GeV/*c*, the kaon efficiency and pion misidentification fraction are found to be ∼95 % and ∼10 %, respectively. The alternative PID requirement of $\Delta \log \mathcal{L}(\mbox{K}-\pi)>5$ illustrates that the misidentification rate can be significantly reduced to ∼3 % for a kaon efficiency of ∼85 %. Figure 18 shows the corresponding efficiencies and misidentification fractions in simulation. In addition to K/*π* separation, both p/*π* and p/K separation are equally vital for a large number of physics analyses at LHCb. Figure 19 demonstrates the separation achievable between protons and pions when imposing the PID requirements $\Delta\mathcal{L}(\mbox{p} -\pi)>0$ and $\Delta\mathcal{L}(\mbox{p} -\pi)>5$. Finally, Fig. 20 shows the discrimination achievable between protons and kaons when imposing the requirements $\Delta\mathcal{L}(\mbox{p} - \mbox{K})>0$ and $\Delta\mathcal{L}(\mbox{p} - \mbox{K})>5$.

Together, Figs. 17, 19 and 20 demonstrate the RICH detectors ability to discriminate any pair of track types, from the set of kaons, pions and protons, albeit for the PID requirements quoted.

### Performance as a function of event multiplicity

The current running conditions,^2^ with increased particle multiplicities, provide an insightful glimpse of the RICH performance at high luminosity running.

**Fig. 21:**
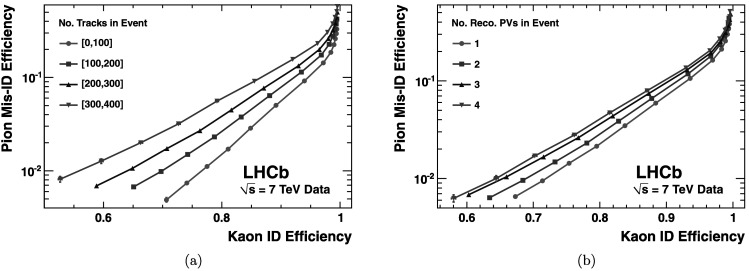
Pion misidentification fraction versus kaon identification efficiency as measured in 7 TeV LHCb collisions: (**a**) as a function of track multiplicity, and (**b**) as a function of the number of reconstructed primary vertices. The efficiencies are averaged over all particle momenta

Figure 21 shows the pion misidentification fraction versus the kaon identification efficiency as a function of (a) track multiplicity and (b) the number of reconstructed primary vertices, as the requirement on the likelihood difference $\Delta \log \mathcal{L}(\mbox{K}-\pi)$ is varied. The results demonstrate, as expected, some degradation in PID performance with increased interaction multiplicity. The K/*π* separation is, however, extremely robust right up to the highest interaction multiplicities and thus gives confidence that the current RICH system is suitable for operation at the higher luminosities foreseen in the future.

## Conclusions

The RICH detector was designed specifically for the physics program of LHCb. It has been in operation since the end of 2009. The RICH detector has operated with high efficiency during these first three years of LHC running. It has demonstrated a PID performance that is well up to design specifications and that allows the extraction of physics results in all sectors of *b* and *c* quark decays, in particular of the rare phenomena which may allow the discovery of new physics at the LHC.

The performance of the RICH particle identification has been studied with the LHC collisions taken since the startup of the LHC machine. Studies of the decays of K^0^, Λ^0^ and D^∗^ provide a source of *π*, K, p identified kinematically for which the RICH identification performance can be established. The precise alignment and calibration procedures are crucial to reach the designed performance. The Cherenkov angle resolutions are in good agreement with the expected design performance for the gas radiators, and are still being improved for the aerogel radiator.

## Footnotes

1 The number of malfunctioning HPDs is considered acceptable if it is less than 6 peripheral tubes, or one central tube.

2 The LHCb RICH detector was designed to run with 0.6 interaction per bunch crossing. However the current operating conditions have 1.6 interactions per bunch crossing.

## References

[1] Alves A.A., LHCb Collaboration (2008). J. Instrum..

[2] Aaij R., LHCb Collaboration (2012). Phys. Rev. Lett..

[3] Aaij R., LHCb Collaboration (2012). Phys. Lett. B.

[4] Aaij R., LHCb Collaboration (2012). Phys. Rev. D.

[5] Aaij R., LHCb Collaboration (2012). Phys. Rev. Lett..

[6] Aaij R., LHCb Collaboration (2012). J. High Energy Phys..

[7] Aaij R., LHCb Collaboration (2012). Eur. Phys. J. C.

[8] R.A. Nobrega et al. (LHCb Collaboration), CERN/LHCC 2003-031, LHCb TDR 10 (2003)

[9] Hesser J.E., Dressler K. (1967). J. Chem. Phys..

[10] Bellunato T. (2004). Nucl. Instrum. Methods A.

[11] Alemi M. (2000). Nucl. Instrum. Methods A.

[12] Eisenhardt S. (2008). Nucl. Instrum. Methods A.

[13] Moritz M. (2004). Performance study of new pixel hybrid photon detector prototypes for the LHCb RICH counters. IEEE Transactions on Nuclear Science.

[14] Papanestis A. (2009). The LHCb RICH detector control system. Proceedings of ICALEPCS.

[15] Fontanelli F. (2009). Nucl. Instrum. Methods A.

[16] Arnaboldi C. (2007). NSS’07 IEEE.

[17] Holme O., Gonzalez Berges M., Golonka P., Schmeling S. (2005). The JCOP framework. Conf. Proc. C051010:WE.

[18] Clemencic M. (2008). J. Phys. Conf. Ser..

[19] Adinolfi M. (2007). Nucl. Instrum. Methods A.

[20] A. Borgia et al. submitted to Nucl. Inst. Methods A. arXiv:1206.0253

[21] Cardinale R. (2011). J. Instrum..

[22] T. Gys, Magnetic field simulations for the LHCb RICH-2 detector. Note LHCb-2002-029

[23] W. Baldini, et al., LHCb alignment strategy. Note LHCb-2006-035

[24] Barrand G. (2001). GAUDI—a software architecture and framework for building HEP data processing applications. Comput. Phys. Commun..

[25] Clemencic M. (2010). Recent developments in the LHCb software framework Gaudi. J. Phys. Conf. Ser..

[26] Forty R. (1996). Nucl. Instrum. Methods A.

[27] Agostinelli S. (2003). Nucl. Instrum. Methods A.

[28] Easo S. (2005). Simulation of LHCb RICH detectors using GEANT4. IEEE Trans. Nucl. Sci..

[29] S. Gorbunov, I. Kisel. CBM-SOFT-note-2005-002

[30] Pivk M., Le Diberder F.R. (2005). Nucl. Instrum. Methods A.

